# Testing an Advocacy Program to Improve Service Access Among Latino Families of Autistic Youth: A Randomized Controlled Trial

**DOI:** 10.1002/aur.70068

**Published:** 2025-06-21

**Authors:** Meghan M. Burke, Saury Ramos‐Torres, Gabriela Herrera Espinosa, Ana Lucia Hincapie, Janeth Aleman‐Tovar, Rocio Perez, Consuelo Puente

**Affiliations:** ^1^ Vanderbilt University Nashville TN USA; ^2^ The University of Texas at Austin Austin TX USA; ^3^ Chico‐School of Education California State University Chico CA USA; ^4^ The Arc of Illinois Tinley Park IL USA

**Keywords:** advocacy, autism, Latino, services

## Abstract

Families of transition‐aged youth with autism often struggle to access services. Due to systemic barriers, Latino, Spanish‐speaking families of autistic youth especially struggle to access services. One way to improve service access is through parent advocacy abilities (i.e., knowledge of adult services, advocacy abilities and comfort, empowerment). To improve parent advocacy abilities and, ultimately, service access, we conducted a randomized controlled trial to test the feasibility and efficacy of an advocacy program: ASISTIR (Apoyando a nueStros hIjo/as con autiSmo obTener servIcios de tRansición; Supporting our Children with Autism to Obtain Transition Services). Of the 30 participants who were retained for analyses, intervention (vs. waitlist‐control) group participants demonstrated significant increases in knowledge about adult services, advocacy activities, advocacy skills and comfort, and empowerment. Further, intervention (vs. waitlist‐control) group participants demonstrated significantly greater service access. Implications for research and practice are discussed.

**Trial Registration:** clinicaltrials.gov: NCT06207149

**Summary**

- It can be hard to find services for autistic youth.
- There are unique barriers to services for Latino autistic youth.
- To improve access to services, we tested an advocacy program (called ASISTIR).
- The ASISTIR program was comprised of 24 h of instruction about adult services.
- Altogether, 30 families participated in the study.
- Some families were randomized to the intervention group, and some families were randomized to the waitlist‐control group.
- Amilies in the intervention group were significantly more likely to be knowledgeable about adult services, comfortable with advocacy, and empowered.
- Intervention group families also reported greater services.

Services are critical in improving outcomes for transition‐aged autistic youth. Over the past decade, autism prevalence estimates have risen dramatically (Harris 2023). Accordingly, an increasing number of autistic youth are exiting high school and entering adulthood. Many autistic youth struggle to obtain employment, community living, and post‐secondary education (Lord et al. 2020). When provided with appropriate services, autistic youth demonstrate significantly improved post‐school outcomes (e.g., greater employment, more likely to live in the community, Alverson and Yamamoto 2017; Scott et al. 2019).

Unfortunately, autistic youth face a “service cliff” when they transition from school‐based to adult service systems. When autistic youth leave high school, they often lose access to services. More than 75% of individuals with disabilities, including autism, do not receive any services after high school exit (Laxman et al. 2019). The decline in services is often attributed to under‐funding of the adult service delivery system (Friedman 2023). However, adult services are difficult to navigate (Anderson et al. 2018). Families must contact different agencies for each service. They must learn the eligibility scheme, bureaucracy, norms, and regulations for each service agency. Then, they must advocate for their youth to prove their eligibility and, once eligible, to access services.

Challenges in service access and, accordingly, post‐school outcomes are exacerbated for Latino, autistic youth and their families. Relative to white youth, Latino youth are less likely to receive long‐term services and supports (Schott et al. 2021), transition services (Eilenberg et al. 2019), and vocational rehabilitation (Roux et al. 2020). Compared to their white autistic peers, Latino youth are less likely to be employed, enrolled in post‐secondary education, and living in the community (Eilenberg et al. 2019). Such poor outcomes can be traced, in part, to service disparities. Indeed, in a study of transition‐aged youth with disabilities, including autism, access to vocational rehabilitative services was more critical to Latino youth (vs. other ethnicities) in retaining employment (Langi and Balcazar 2017).

Despite such disparities, few interventions are targeted for Latino, transition‐aged autistic youth and their families; none of the extant interventions aim to increase access to adult services. Most interventions for autistic youth are developed and tested with Euro‐American cultural norms (Rios and Burke 2021). When interventions are culturally adapted, they are significantly more effective than non‐adapted interventions (Cardona et al. 2012). Of the extant evidence‐based interventions for families of transition‐aged autistic youth, only one intervention (*Juntos en la Transición*) is available in Spanish and is culturally informed for Latino families (Kuhn et al. 2020). However, *Juntos en la Transición* is focused on improving parent well‐being, parent–child relationships, and youth social interactions. *Juntos en la Transición* does not aim to increase access to services.

In the proposed project, we tested the feasibility and effectiveness of a culturally responsive intervention (called ASISTIR or Apoyando a nueStros hIjo/as con autiSmo obTener servIcios de tRansición; Supporting our Children with Autism to Obtain Transition Services). It is important to ensure that an intervention is feasible (i.e., will be attended by the targeted population); if an intervention is not feasible, then its effectiveness does not matter (Goddard and Harding 2003). With respect to effectiveness, prior research suggests that parent advocacy abilities (i.e., knowledge of adult services, advocacy skills, and empowerment) are mechanisms of change to impact service access (e.g., Burke et al. 2019; Casagrande and Ingersoll 2021; Taylor et al. 2017). Such parent advocacy abilities may be especially helpful among families of youth with autism as the abilities are applicable to families of autistic youth across a range of functioning. Thus, we tested whether ASISTIR was effective in improving parent advocacy abilities and, subsequently, service access.

The research questions for this study were: (1) What is the feasibility (i.e., attendance, attrition, and participant satisfaction) of ASISTIR?; (2) What are the proximal effects of ASISTIR on parent advocacy abilities: knowledge of adult services, advocacy, and empowerment?; and (3) What are the distal effects of ASISTIR on service access (i.e., service receipt and unmet service needs)? In alignment with prior research about advocacy programs for Latino families of autistic youth (e.g., Rios and Burke 2023), we hypothesized that attendance would be greater than 70%, attrition would be less than 15%, and that greater than 80% of participants would report high satisfaction with the ASISTIR program. With respect to proximal effects, consistent with extant research about advocacy programs (e.g., Taylor et al. 2017), we hypothesized that the intervention (vs. waitlist‐control group) would report significantly greater knowledge, advocacy, and empowerment. Further, we hypothesized that, six months after beginning the ASISTIR intervention, the intervention (vs. waitlist‐control group) would demonstrate greater access to services and fewer unmet service needs.

## 1 Method

### 1.1 Participants

To be included in the study, participants needed to: have a child older than the age of 12 with autism; identify as Latino; live in a Midwestern state; and speak Spanish. Participants could be bilingual in English and Spanish; however, because the ASISTIR program was delivered in Spanish, they must speak Spanish. The age of 12 was chosen as prior research suggests that families want to learn about adult services as early as the age of 12 (Francis et al. 2018). Both parent report and scores on the Social Responsiveness Scale (SRS, Constantino and Scale 2012) were used to confirm an autism diagnosis.

**FIGURE 1:**
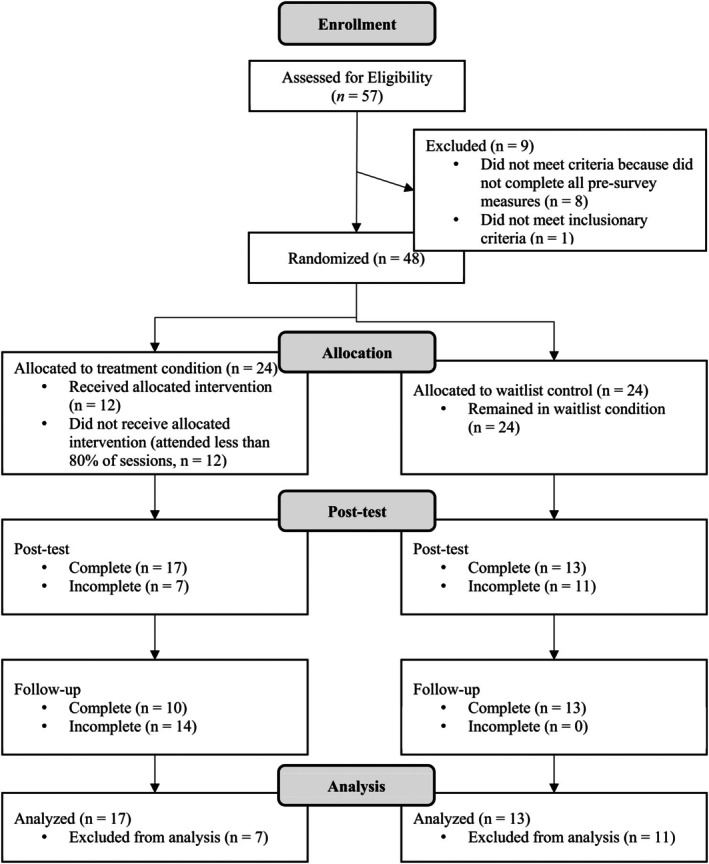
CONSORT flowchart.

As shown in Figure 1, a total of 57 participants were initially recruited for the project. However, nine individuals did not meet the inclusionary criteria. The remaining 48 participants were randomized; specifically, 24 participants were randomized to the intervention group and 24 participants to the waitlist‐control group. Of the 48 randomized participants, 30 (62.50%) were retained for the analyses; the remaining 18 participants were lost to follow‐up. There were no significant demographic differences between the intervention and waitlist‐control groups. See Table 1 for participant demographics across intervention and waitlist‐control groups. Before conducting the study, an a priori power analysis was conducted based on the effect size (*f* ^2^ = 0.45) derived from a previous advocacy program (Taylor et al. 2017). Using traditional assumptions (e.g., power = 80%, *p* < 0.05, attrition rate was 10%), the power analysis indicated that 40 participants were needed for a fully powered study; thus, this study is underpowered to detect significant differences due to participants being lost to follow‐up.

**TABLE 1:** Participant demographics.

	Intervention (*n* = 24)	Control (*n* = 24)	*t*/*X* ^2^	*p*
Participant age	45.75 (9.35)	44.72 (6.12)	−0.46	0.33
Participant gender: Female	75.0% (18)	95.83% (23)	2.33	0.18
Participant educational background			−1.58	0.06
Less than high school degree	20.83% (5)	29.17% (7)		
Received high school degree	25.0% (6)	33.33% (8)		
Some college	20.83% (5)	29.17% (7)		
Associate's degree	0% (0)	0% (0)		
Bachelor's degree	29.17% (7)	8.33% (2)		
Master's degree	4.17% (1)	0% (0)		
Participant annual household income			−1.13	0.13
Less than $20,000	16.67% (4)	37.50% (9)		
$20,001–$40,000	20.83% (5)	20.83% (5)		
$40,001–$60,000	41.67% (10)	29.17% (7)		
$60,001–$80,000	8.33% (2)	4.17% (1)		
$80,001–$100,000	8.33% (2)	8.33% (2)		
Prefer not to answer	4.17% (1)	0% (0)		
Youth age	16.57 (6.38)	14.26 (5.10)	−1.33	0.09
Youth gender: Male	87.50% (21)	95.83% (23)	0.29	0.59

### 1.2 Recruitment

Recruitment was conducted in several ways. We partnered with community‐based disability agencies in a Midwestern state that served Latino families to recruit participants. The agencies used personalismo (i.e., personal relationships and friendliness, Magaña 2000), word of mouth, recruitment flyers, and social media to share information about the study. All recruitment materials were available in English and Spanish. Participants received a $20 gift card at the post survey and a $20 gift card at the follow‐up survey.

### 1.3 Compliance With Ethical Standards

We received Institutional Review Board (IRB) approval for this project. We have no conflicts of interest. All research involving participants provided informed consent to be included in this study.

### 1.4 Procedures

First, this study was registered at clinicaltrials.gov (NCT06207149). After providing consent and completing the baseline survey, participants were randomly assigned to the intervention or waitlist‐control group by the study coordinator. Specifically, participants were randomly assigned using 1:1 simple randomization that was generated by a computer software program. Intervention group participants took ASISTIR during the fall of 2023; waitlist‐control group participants took ASISTIR in spring of 2024. The study coordinator shared the randomization results with each participant. At the beginning of each ASISTIR session, a research assistant took attendance data. At the end of each ASISTIR session, each participant completed a formative evaluation to gauge their satisfaction with the session. At the end of the intervention group completing the ASISTIR program, all participants were asked to complete the post survey. Six months after the intervention group started the ASISTIR program, all participants were asked to complete the follow‐up survey. Prior research has suggested that 6 months may be a sufficient amount of time to see an effect on service access (Taylor et al. 2017). At the six‐month mark and after completing the follow‐up survey, all waitlist‐control group participants were invited to participate in the ASISTIR program. All surveys were completed online via RedCap.

### 1.5 The ASISTIR Program

The ASISTIR program was comprised of 24 h of content about adult services. The program was conducted via zoom. Notably, the community‐based organization suggested that conducting ASISTIR via zoom (vs. in‐person) would yield a larger sample. The ASISTIR program content was developed with input from Latino parents of children with autism (Aleman‐Tovar et al. 2023). The first 12 h of the ASISTIR program were pilot‐tested with 29 Latino parents of autistic youth (Aleman‐Tovar et al. 2025). The ASISTIR program was co‐facilitated by two Latina mothers of adult children with autism; both mothers worked at a statewide community‐based disability organization. The facilitators introduced the content and speaker, addressed logistical challenges during the session (as needed), shared resources, and attended each session. Each session lasted 2 h; sessions were conducted in a group format on a weekly basis. It takes 12 weeks to complete the program. Topics included: person‐centered planning, secondary education, models of decision‐making, Supplementary Security Income (SSI), Social Security Disability Insurance (SSDI), Supplemental Nutrition Assistance Program, health insurance, Medicaid Home and Community‐Based Services (HCBS) waivers, Vocational Rehabilitative Services, Housing, Technology, Special Needs Trusts, and Achieving A Better Life Experience (ABLE) accounts, post‐secondary education, and advocacy.

At the beginning of each session, the participants watched a video introducing them to the content. In the video, a Latina mother of an autistic youth shared information about the relevant topic. The video also included a family story of the experience of a Latino family with the topic. Then, the facilitator introduced a local expert. The expert was an individual who had expertise in the topic at hand; experts reflected various organizations (e.g., the Parent Training and Information Center [PTI], the University Center for Excellence in Developmental Disabilities, the Social Security Administration). With the exception of one expert, all experts identified as Latino and were native Spanish‐speakers. We could not find an expert in housing who also identified as Latino and spoke Spanish; accordingly, the expert on housing gave her presentation in English with the research team conducting automatic translation into Spanish. The facilitators were from a community‐based organization that provided support to Latino families via trainings, information and referral, and individualized assistance.

### 1.6 Facilitator Fidelity to the ASISTIR Program

At each session, a research assistant collected facilitator fidelity data using a pre‐established checklist. The checklist included items related to program content (e.g., did the facilitator review the topic from last week?). If an item was covered during the program, it was marked as having fidelity. Fidelity to the curriculum was 87.76%. A second research assistant independently collected fidelity data for 50% of sessions; their inter‐rater agreement was 93.18%. All disagreements were discussed between the coders until they reached consensus.

### 1.7 Measures

All measures were available in English and Spanish to the participants. Regarding the latter, each measure had been forward and back translated in prior studies and tested for reliability and validity with Spanish‐speaking, Latino families of autistic youth (Aleman‐Tovar et al. 2023). The majority of the measures were completed in Spanish.

#### 1.7.1 Measure of Feasibility: Attendance and Attrition

Attendance was calculated as the percentage of the total number of sessions attended for each participant divided by the total number of sessions (i.e., twelve). Attrition was gauged by the overall completion of the ASISTIR program. Specifically, completion was calculated as the number of individuals who completed the entire ASISTIR program divided by those who attended the first session.

#### 1.7.2 Measure of Feasibility: Formative Evaluations

The formative evaluation had been used in prior studies about advocacy (e.g., Burke et al. 2016; Rios and Burke 2023; Taylor et al. 2022). The formative evaluation included nine close and open‐ended questions about the session. For example, close‐ended questions were about the clarity of the presentation, its relevance, and participant satisfaction. Open‐ended questions included items aboutwhat was most helpful about each session and suggestions for improvement. All formative evaluations were anonymous.

#### 1.7.3 Measure of Proximal Effectiveness: Adult Disability Services Knowledge

We asked 22 multiple choice questions about special education knowledge (Taylor et al. 2017, 2023). A sample item was “Which of the following requires person‐centered planning (i.e., wherein all decisions are based on the strengths, needs, and preferences of the person with a disability)?” Each question had four response options; only one response option was correct. One of the response options was “I do not know.” For this study, items were summed into a composite score with higher scores indicating a greater number of correct responses.

#### 1.7.4 Measure of Proximal Effectiveness: Parent Advocacy Activities

Comprised of 15 items reflecting individual, peer, and systemic advocacy, each item of the Advocacy Activities Scale (Li et al. 2024) has a 5‐point Likert scale from (1) *not at all* to (5) *very often*. For this study, only individual advocacy activities were expected to increase among the intervention group. A sample item for individual advocacy was: “Called agencies to ask about services”. Higher scores indicate greater frequency of advocacy activities. Reliability was high (*α* = 0.86 for the Individual Advocacy Subscale).

#### 1.7.5 Measure of Proximal Effectiveness: Advocacy Skills and Comfort

We used the 10‐item Advocacy Skills and Comfort Scale (Burke et al. 2016) to examine knowledge and comfort with advocacy. A sample item was “How able are you to effectively communicate with providers/agencies/professionals?” Response options included: (1) *Not at all*, (2) *Below average*, (3) *Average*, (4) *Good*, and (5) *Excellent*. Higher scores indicate greater comfort with advocacy skills. Reliability was high (*α* = 0.91).

#### 1.7.6 Measure of Proximal Effectiveness: Family Empowerment

The 34‐item Family Empowerment Scale (Koren et al. 1992) measures empowerment across three subscales: Family, the Service Delivery System, and the Community/Political System. A sample item was “When problems arise with my child, I handle them pretty well.” Items are rated on a 5‐point Likert scale from (1) *not at all true* to (5) *very true*. Higher scores indicate greater empowerment. Reliability was high (*α*'s = 0.88) for the Family Empowerment Subscale, 0.91 for the Service Delivery System Subscale, and 0.80 for the Community/Political Subscale.

#### 1.7.7 Measure of Distal Effectiveness: Service Access

Service Access was measured via the Services Inventory (Burke and Fulton 2023). Specifically, participants were asked about their receipt of 10 governmental programs that provide services: SSI, SSDI, Vocational Rehabilitative Services, Medicaid HCBS Waivers, Medicaid Long Term Services and Supports, Guardianship/Conservatorship, Special Needs Trust and/or ABLE Accounts, Housing Choice Vouchers, SNAP, and Medicaid or Medicare Health Insurance. Participants answered whether or not they received each service: (0) *No*; (1) *Yes*. If they did not receive the service, participants were asked whether they needed the service: (0) *No*; (1) *Yes*. Service receipt was calculated as the sum of the services the participant received; unmet service needs were calculated as the sum of the services needed but not received.

### 1.8 Analysis

For the first research question about feasibility, we conducted descriptive statistics of the close‐ended questions. For the open‐ended questions, we conducted open coding (Creswell 2003) and a frequency count of themes. Notably, we did not have a priori codes for the data. We used a word‐by‐word approach to analyze the qualitative data. We reviewed the codes several times. After reaching consensus on the codes, we grouped the codes into categories and clustered the categories into themes. For the second research question about proximal effectiveness, we conducted a MANCOVA, controlling for baseline scores, to discern differences in the post scores on knowledge of adult disability services, advocacy skills and comfort, advocacy activities, and empowerment. Given the multitude of tests, a Bonferroni correction was used suggesting that *p*'s < 0.01 were significant. For the third research question about distal effectiveness, we conducted a non‐parametric, repeated measures ANCOVA by condition. The dependent variables were the number of services received and unmet service needs.

## 2 Results

### 2.1 Feasibility of ASISTIR

Average attendance across all 12 sessions was 67.5% with an attrition rate of 0%. Specifically, 11 participants attended the first session; this was the lowest attended session. All 11 participants who attended the first session attended the remaining sessions. For other sessions, attendance ranged from 58.33% (*n* = 14) to 83.33% (*n* = 20). Reasons for not attending included challenges with finding childcare and conflicting work schedules.

With respect to the formative evaluation feedback, overall, participants were satisfied with ASISTIR. Across all of the sessions except for one (health insurance), more than 60% of participants reported the speaker had “excellent” knowledge of the given topic. For all sessions, at least 60% of the participants reported the topics were “relevant.” With very few exceptions, the participants overwhelmingly reported that the sessions were of “appropriate length.” With respect to satisfaction, for each session, more than 60% of participants reported “high satisfaction.” See Table 2.

**TABLE 2:** Formative evaluation data.

Session^a^	1 (*n* = 8)	2 (*n* = 16)	3 (*n* = 9)	4 (*n* = 16)	5 (*n* = 17)	6 (*n* = 13)	7 (*n* = 13)	8 (*n* = 15)	9 (*n* = 16)	10 (*n* = 15)	11 (*n* = 13)	12 (*n* = 12)
Quality of the presentation												
Excellent	75% (6)	87.5% (14)	88.89% (8)	68.75% (11)	76.47% (13)	46.15% (6)	92.31% (12)	73.33% (11)	62.5% (10)	73.33% (11)	84.62% (11)	83.33% (10)
Good	25% (2)	12.5% (2)	11.11% (1)	31.25% (5)	23.53% (4)	53.85% (7)	7.69% (1)	20% (3)	37.5% (6)	26.67% (4)	15.38% (2)	16.67% (2)
Average	—	—	—	—	—	—	—	6.67% (1)	—	—	—	—
Clarity of the presentation												
Excellent	62.5% (5)	75% (12)	77.78% (7)	75% (12)	76.47% (13)	61.54% (8)	84.62% (11)	73.33% (11)	62.5% (10)	80% (12)	84.62% (11)	83.33% (10)
Good	25% (2)	25% (4)	22.22% (2)	25% (4)	23.53% (4)	38.46% (5)	15.38% (2)	26.67% (4)	31.25% (5)	20% (3)	15.38% (2)	16.67% (2)
Average	12.5% (1)	6.25% (1)	—	—	—	—	—	—	6.25% (1)	—	—	—
Relevance of the topic of the presentation												
Excellent	62.5% (6)	81.25% (13)	66.67% (6)	62.5% (10)	76.47% (13)	76.92% (10)	84.62% (11)	73.33% (11)	68.75% (11)	80% (12)	76.92% (10)	83.33% (10)
Good	25% (2)	12.5% (2)	33.33% (3)	37.5% (6)	23.53% (4)	23.08% (3)	15.38% (2)	26.67% (4)	31.25% (5)	13.33% (2)	23.08% (3)	16.67% (2)
Average	12.5% (1)	6.25% (1)	—	—	—	—	—	—	—	6.67% (1)	—	—
Session length												
Too long	—	—	—	—	—	—	—	13.33% (2)	—	—	—	—
Just right	100% (8)	100% (16)	100% (9)	100% (16)	100% (17)	100% (13)	92.31% (12)	73.33% (11)	100% (16)	100% (15)	92.31% (12)	100% (12)
Too short	—	—	—	—	—	—	7.69% (1)	13.33% (2)	—	—	7.69% (1)	—
Participant satisfaction												
High	87.5% (7)	75% (12)	88.89% (8)	81.25% (13)	82.35% (14)	61.54% (8)	100% (13)	86.67% (13)	68.75% (11)	80% (12)	84.62% (11)	83.33% (10)
Average	12.5% (1)	25% (4)	11.11% (1)	18.75% (3)	17.65% (3)	38.46% (5)	—	13.33% (2)	31.25% (5)	20% (3)	15.38% (2)	16.67% (2)

^a^ Session 1: Introduction to ASISTIR; Session 2: SSI and SSDI; Session 3: Decision‐making options; Session 4: Secondary education and transition; Session 5: Vocational Rehabilitative Services; Session 6: Health insurance; Session 7: Medicaid waivers; Session 8: Assistive technology and housing; Session 9: Post‐secondary education; Session 10: SNAP and other governmental benefits; Session 11: Special needs trusts; and Session 12: Advocacy.

In response to “What helped you the most?” (Lo que más me sirvió), participant responses clustered around three themes: everything, applying for specific services, and learning ways to promote independence for autistic youth. Regarding “everything,” some participants simply wrote that all of the content was helpful. A participant wrote, “Everything because I am new to these topics” (Todo porque soy nueva en estos temas). Another participant wrote, “As always, everything has helped me” (Como siempre, todo me ha ayudado).

Perhaps in contrast to the participants who reported that “everything” in ASISTIR was helpful, some participants cited specific content about certain services was helpful. However, there was no uniformity about which service was most helpful. Services included: insurance, school, social security, guardianship, housing, and special needs trusts. As an illustration of the varied responses, here is a subsample of their written responses: “The ability to be sure that my son will be protected through a trust, even after my passing” (El poder estar segura de que mi hijo estará protegido por medio de un fideicomiso, aun después de mi fallecimiento); “Knowing that there will be a place where my son could live” (Saber que habrá un lugar en donde mi hijo podría vivir); “Knowing where and who to contact when I have questions about Social Security” (Saber dónde comunicarme cuando tengo dudas acerca del seguro social); and “The information about insurance and how to apply it to get help for our children” (La información acerca del seguro y como aplicarla para conseguir ayuda para nuestros hijos).

Finally, some responses related to learning ways to include autistic youth in advocacy and to promote the youth's independence. A participant wrote, “Knowing that we have options for our children to become independent and that they have different housing options where they can thrive” (Saber que tenemos opciones para que nuestros hijos puedan hacerse independientes y que tienen diferentes opciones de vivienda en donde florecer). Another participant wrote, “Knowing that my son can be present at IEP meetings and that he can have a say about his future” (Saber que mi hijo puede estar presente en las juntas del IEP y que puede opinar acerca de su futuro).

With respect to suggestions to improve ASISTIR, the overwhelming response was the need for more time for participants to speak to one another and ask and answer questions. Regarding the former, some participants reported that they wanted more time to share their experiences so they could learn from their peers. Succinctly written by a participant: “Allow participants to express their experiences so that we can learn from them as well” (Permitir que los participantes expresen sus experiencias para así poder aprender de ellas también). Some participants also wanted more time—but specified that the time should be for questions and answers. Such responses included: “Everything was perfect, but time flew by and there wasn't enough time for quality questions and answers” (Todo fue perfecto, pero el tiempo se pasó volando y no dio el tiempo suficiente para preguntas y respuesta de calidad) and “I would like the section to be divided into two parts to be able to ask questions instead of waiting until the end. The time went by very quickly, and the time for questions was short” (Me gustaría que la sección se pudiera dividir en dos partes para poder hacer preguntas y no esperar hasta el final. el tiempo se pasó muy rápido y el tiempo para preguntas fue corto).

### 2.2 Proximal Effects of ASISTIR

With respect to proximal effects on knowledge about adult disability services, individual advocacy activities, advocacy skills and comfort, and the three subscales of the Family Empowerment Scale (i.e., family empowerment subscale, service delivery system subscale, and the community/political empowerment subscale), the overall model was significant, *F* (5, 30) = 4.34, *p* < 0.01. Specifically, the intervention (vs. waitlist‐control group) demonstrated significant improvements with respect to: knowledge of disability services (*p* = 0.02, ES = 0.28), individual advocacy activities (*p* = 0.01, ES = 0.31), advocacy skills and comfort (*p* = 0.03, ES = 0.25), and community/political empowerment (*p* = 0.02, ES = 0.27). Although there were increases among the intervention (vs. control group), there were no significant differences with respect to family empowerment and service delivery system empowerment. See Table 3.

**TABLE 3:** Pre, post, follow‐up survey differences.

Variable	Control (*n* = 13)	Intervention (*n* = 17)
	*X̄* (SD)	*X̄* (SD)
Adult disability services knowledge		
Baseline	4.16 (3.27)	5.71 (4.35)
Post‐intervention*	3.68 (4.59)	10.62 (2.57)
Follow‐up	3.04 (3.45)	6.84 (4.23)
Individual advocacy activities		
Baseline	16.21 (3.55)	18.50 (6.05)
Post‐intervention*	15.61 (5.28)	20.00 (5.77)
Follow‐up	16.69 (4.63)	33.75 (7.49)
Advocacy skills and comfort		
Baseline	28.21 (6.40)	26.72 (8.35)
Post‐intervention*	25.38 (9.51)	33.92 (8.42)
Follow‐up	29.23 (9.01)	33.75 (7.49)
Family empowerment subscale		
Baseline	44.21 (7.15)	47.27 (7.69)
Post‐intervention	41.07 (10.48)	44.58 (0.95)
Follow‐up	46.62 (9.38)	51.38 (8.36)
Service delivery system empowerment subscale		
Baseline	43.89 (7.78)	46.54 (8.84)
Post‐Intervention	42.31 (11.16)	47.31 (5.89)
Follow‐up	46.61 (9.38)	51.00 (8.73)
Community/political empowerment subscale		
Baseline	29.94 (6.78)	30.72 (7.25)
Post‐intervention*	29.62 (8.45)	37.23 (7.13)
Follow‐up	33.31 (5.83)	37.50 (7.25)

*p < 0.01.

### 2.3 Distal Effects of ASISTIR

With respect to distal effects of ASISTIR on service receipt, there were significant differences between the groups (Table 4). The model for number of services received was significant for the main effect of group (*F* = 8.61, *p* = 0.004, partial eta squared = 0.55). Specifically, among the waitlist‐control group, there were no significant differences across pre, post, and follow‐up scores for services received. However, for the intervention group, there was a significant increase at the post survey in the number of services received (*p* = 0.04) and a significant increase from the post survey to the follow‐up survey (*p* < 0.01). Altogether, at follow‐up, intervention (vs. waitlist‐control) group participants received, on average, one more service. For unmet service needs, while there were differences between the intervention and waitlist‐control groups, these differences were not significant (*F* = 1.04, *p* = ns). See Table 4.

**TABLE 4:** Pre, post, follow‐up comparisons for service access.

	Control (*n* = 10)	Intervention (*n* = 13)
	*X̄* (SD)	*X̄* (SD)
Number of services received*		
Baseline	1.00 (1.19)	0.55 (0.88)
Post‐intervention	1.88 (1.72)	1.78 (1.92)
Follow‐up	1.50 (1.51)	2.56 (1.67)
Unmet service needs		
Baseline	1.63 (1.69)	1.33 (2.24)
Post‐intervention	0.75 (1.49)	1.00 (3.00)
Follow‐up	3.12 (1.80)	1.89 (1.96)

*p < 0.01.

## 3 Discussion

Compared to white families, Latino, Spanish‐speaking families face systemic and exacerbated barriers in accessing services. Few interventions exist in Spanish that target Latino families of transition‐aged youth with autism to access adult services. Thus, this study contributes to the literature by testing an advocacy program for Latino, Spanish‐speaking families of autistic, transition‐aged youth. There were three main findings. First, overall, the ASISTIR program was feasible and effective. Regarding the former, consistent with prior advocacy programs for Latino families (Rios and Burke 2023), attendance was nearly 70%, attrition was low (0%), and participant satisfaction was high (greater than 80%). Feasibility is important because, if a program is not feasible for participants to attend, it may not matter whether it is effective (Goddard and Harding 2003). The feasibility of this program is especially important given the formal constraints facing most participants (e.g., most participants reported low‐incomes and/or limited formal educational backgrounds). Most research about families of youth with autism reflects white, well‐resourced, and highly educated samples (Lee and Meadan 2021; Steinbrenner et al. 2022). This study extends the literature by reflecting that families from low‐resourced backgrounds can and do participate in longer interventions.

Relatedly, while the sample was primarily low‐resourced with respect to income and formal education, the participants had other forms of capital relevant to this study. In alignment with Yosso's community cultural wealth framework (Yosso 2005), participants seemed to have tremendous family and social capital. For example, on the family empowerment subscale, participants, on average, reported scores around 44 at baseline and 47 at the post survey; the highest score possible is a 60. Thus, on average, most participants were in the top quartile of the subscale reflecting tremendous familial capital. This finding aligns with extant research about the role of familismo in Latino culture (i.e., putting the collective needs of the family ahead of an individual, Steidel and Contreras 2003). With respect to social capital, the importance of talking with one another was a clear theme in the formative evaluations. Altogether, these findings suggest that strengths‐based interventions should leverage the familial and social capital of Latino families.

Second, the program was effective. Intervention (vs. waitlist‐control) group participants demonstrated significant increases in knowledge, individual advocacy activities, skills, and comfort with advocacy, and community/political empowerment. This finding aligns with prior research about advocacy programs finding that such programs can lead to improved parent advocacy abilities (e.g., Rios and Burke 2023; Taylor et al. 2017; Taylor et al. 2023). The formative evaluation findings further support that the ASISTIR program was effective. Many participants cited that either the entire ASISTIR content or specific aspects of the program were helpful to them. Altogether, this finding suggests that ASISTIR can be effective in immediately improving advocacy abilities among Latino, Spanish‐speaking parents of autistic youth.

A next step may be to consider how dosage impacts changes in proximal effects. In a review of the literature about advocacy programs, Rios and Burke (2021) found that most programs were 12 h in duration. ASISTIR is much longer in duration. On the one hand, feasibility was high, suggesting that ASISTIR was accessible for many families. Further, several participants requested more time so they could ask questions and talk with one another. Conversely, families in general are often busy. Families of autistic youth are especially busy (Smith et al. 2010). To improve the feasibility of family interventions, there have been an increasing number of low‐intensity interventions for families of children with autism (Luelmo et al. 2021). Research is needed to understand the tradeoff between feasibility (with respect to duration of the intervention) and effectiveness.

Third, ASISTIR may lead to significant increases in the number of services received by the youth. Specifically, in this study, intervention group participants received, on average, one additional service by the follow‐up timepoint. This finding further underscores the effectiveness of the ASISTIR program, suggesting it could influence family and youth outcomes. Further, the increase at the final timepoint suggests that it could take time to see an effect on service access. Indeed, it can be time‐consuming to identify, apply for, qualify for, and receive a service (Laxman et al. 2019). This finding not only suggests that ASISTIR may be effective but also that the distal effect may take time to impact youth outcomes. This finding may be especially salient for other service interventions in trying to identify the data collection timepoints wherein one could expect to see an impact on service access.

While it is exciting that there was an improvement in service access, future research may consider exploring the specific ways in which ASISTIR may impact service receipt. It is important to beyond the receipt of a service to consider other dimensions of service access. Such dimensions may include: is the service provider a fit with the autistic youth?; is the service duration appropriate?; does the service reflect evidence‐based practice?; and is modality of the service appropriate? (Burke and Taylor 2023). By using a more fine‐grained analysis of service access, we can better discern the effect of the ASISTIR program (and other interventions) on improving service outcomes for autistic youth.

While an important launching point, there are limitations to this study. First, this study reflects individuals who were interested in participating in an advocacy program; the findings may not be generalizable to individuals who may have less time or resources to attend an advocacy program. Perhaps relatedly, it may be that the participants already received some guidance from the community‐based organization prior to the study. Second, this study was slightly underpowered to detect effects. Future research requires a larger sample to meaningfully determine the effects of the ASISTIR intervention. Also, with a larger sample, future research could discern which services are more attainable after completing ASISTIR. Third, the data collection was primarily parent report; other measures may be helpful to have a holistic understanding of the effect of the intervention. Finally, while facilitator fidelity to the program was high, no measure was used for fidelity for the participants.

### 3.1 Implications for Research and Practice

Future research should include other aspects that impact service delivery. In a review of factors that contribute to service disparities among Latino families, several barriers were identified, including: language barriers, racism, classism, limited cultural matching between families and providers, and long waiting lists (Smith et al. 2020). Understanding whether ASISTIR mitigates such barriers to service delivery can help shed light on the mechanisms through which ASISTIR may improve service access. Such research also aligns with a recent call to increase research about health equity among autistic individuals (Straiton et al., 2024).

Research is also needed that reflects the perspective of the autistic youth. Transition planning hinges on the participation of the youth with a disability—this includes choices, advocacy, and decision‐making about adult disability services (Shogren and Raley 2022). The formative findings from this study suggest that there was an impact on independence and self‐determination of the youth. Thus, future research should include the autistic youth in the intervention and in data collection.

There are also implications for practice. In every state in the United States, there is a (PTI), which is federally funded to educate and empower families of children with disabilities (aged 0–26). PTIs are especially charged with supporting families who are marginalized (e.g., Latino, Spanish‐speaking families). PTIs may consider replicating the ASISTIR program with their constituencies to educate and empower them to access adult services. As PTIs consider replicating the ASISTIR program, practitioners may consider some of the formative evaluation findings. Specifically, practitioners may reflect on the importance of Latino families learning from one another, offering the program in Spanish, and primarily relying on Latino, Spanish‐speaking presenters to facilitate the training. Further, practitioners may consider strategies to enable Latino families to meet one another. To that end, they may consider embedding more group chat strategies (e.g., Whatsapp or Telegram) so participants can meet one another. Also, given that advocacy for Latino families is an international phenomenon (Cohen 2013), practitioners in other countries may consider replicating aspects of ASISTIR to help Latino families advocate.

## Conflicts of Interest

The authors declare no conflicts of interest.

## Supporting information


**Data S1.** CONSORT‐2010‐Checklist.

## Data Availability

The data that support the findings of this study are openly available in NDAR at https://healthdata.gov/widgets/7ue5‐z77y?mobile_redirect=true.
